# Early prenatal diagnosis of an atypical phenotype of sacral spina bifida 

**DOI:** 10.25122/jml-2021-0292

**Published:** 2021

**Authors:** Roxana Elena Bohîlțea, Bianca Margareta Mihai, Octavian Munteanu, Ioniță Ducu, Vasile Adrian Dumitru, Consuela-Mădălina Gheorghe, Tiberiu Augustin Georgescu, Valentin Varlas, Radu Vlădăreanu

**Affiliations:** 1. Department of Obstetrics and Gynecology, Carol Davila University of Medicine and Pharmacy, Bucharest, Romania; 2. Department of Obstetrics and Gynecology, Filantropia Clinical Hospital, Bucharest, Romania; 3. Department of Obstetrics and Gynecology, University Emergency Hospital, Bucharest, Bucharest, Romania; 4. Department of Pathology, Carol Davila University of Medicine and Pharmacy, Bucharest, Romania; 5. Department of Marketing and Medical Technology, Carol Davila University of Medicine and Pharmacy, Bucharest, Romania; 6. Department of Obstetrics and Gynecology, Elias University Emergency Hospital, Bucharest, Romania

**Keywords:** neural tube defects, embryogenesis, ultrasonographic diagnosis, meningocele, SARS-COV 2 maternal infection, NTDs – neural tube defects, 2D ultrasonography – two-dimensional ultrasonography, SARS-COV 2 – severe acute respiratory syndrome coronavirus 2

## Abstract

Neural tube defects (NTDs) occur during embryogenesis, specifically during the fifth or sixth week of gestation, and are described as aberrant neural tube closing. The defect may alter the normal development of the vertebrae, spinal cord, cranium, or brain. The present study describes the case of a 41-year-old pregnant woman with fetal sacral meningocele, no associated pathologies, no family history of neural tube defects, a pregnancy under folate supplementation with the aim of highlighting the importance of ultrasound in diagnosing neural tube defects. The ultrasonographic diagnosis was not clear from the beginning. In our case, the differential diagnosis of meningocele was made with the cystic compound of a sacrococcygeal teratoma, which represents one of the most common congenital tumors in newborns. The particularity of this case was that a neural tube defect occurred despite the prophylactic administration of folic acid during pregnancy, which represents a well-documented protection against neural tube defects in fetuses.

## Introduction

Neural tube defects (NTDs) occur during embryogenesis, specifically during the fifth or sixth week of gestation, and are described as aberrant neural tube closing. The defect may alter the normal development of the vertebrae, spinal cord, cranium, or brain [1]. The incidence is estimated between 0.5 to 8 cases per 1000 births [2]. Spinal dysraphism includes open or closed spinal defects, and the defect may be covered by a membrane or not, the nervous tissue being exposed to the environment [3]. Open neural tube defects do not present a membrane or skin covering the defect and include myelomeningocele and myelocele. Closed neural tube defects are covered by skin and could present a subcutaneous mass. Spinal dysraphism without a subcutaneous mass present can be complex, from split cord malformation to caudal regression syndrome or simple, including filar lipoma, dermal sinus, or tight filum terminale. If the subcutaneous mass is detected, the differential diagnosis must be made between myelocystocele, lipomyeloschisis, lipomyelomeningocele, and meningocele [3].

Concerning etiopathogenesis, the maternal risk factors for neural tube defects include folic acid deficiency, maternal obesity, gestational diabetes, and drugs administered to the mother. The advantage of folic acid supplementation during the conception and first part of the pregnancy has been well documented. Folic acid deficiency caused by folic acid antagonists, dietary deficiency, or genetic abnormalities that interfere with the folate metabolism has been correlated with neural tube defects [4, 5]. Obese mothers have a twofold higher risk of delivering a newborn with spina bifida than normal-weight mothers [6, 7]. Although a mechanism for caudal regression syndrome has not yet been described, it presents a higher incidence in patients with gestational diabetes [8, 9]. Caudal regression syndrome in newborns of diabetic mothers has an incidence of 1–2 cases per 1000 since, in the general population, the incidence is 0.05–0.10 per 1000 [10]. Regarding drug administration, an increased risk for the development of neural tube defects in fetuses whose mothers have received antiseizure drugs such as valproate or carbamazepine during pregnancy has been reported [11, 12].

A meningocele is a closed neural tube defect, a cerebrospinal fluid-filled sac covered by skin, which does not contain a spinal cord protruding through the spinal defect. Usually, the spinal cord and spinal nerves are not affected, although cord tethering may appear. Meningoceles can be posterior or anterior; the posterior location is more frequent. Meningoceles can be localized in the cervical, thoracic, lumbar, or sacral spinal; the most common location is in the lumbar or sacral spine [13, 14]. In a recently published article, Toru *et al.* [15] reported meningocele as a constitutive element of diverse syndromes such as Meckel-Gruber syndrome, Schisis association, and Caudal regression syndrome, being associated with the following malformations: unilateral renal agenesis, anal atresia, unilateral diffuse cystic dysplasia of kidney, absent uterus and vagina, bilateral congenital pulmonary adenomatoid malformation type 3, omphalocele, pelvicalyceal dilatation, internal hydrocephalus, congenital diaphragmatic hernia, unilateral renal and adrenal gland agenesia, polydactyly, fusion of adrenal glands, polycystic kidneys, bifid costa, facial cleft [16] and unilateral upper extremity amputation. The diagnosis of a meningocele is formulated using ultrasonography, being widely available and having a low cost. A 100% accuracy has been reported in experienced centers [17]. Spinal abnormalities and associated central nervous system or non-central nervous system abnormalities have been diagnosed using ultrasonography with a 100% specificity rate and 97% sensitivity rate, ultrasonography being extremely helpful in evaluating fetal growth and fetal well-being [18, 19].

The present study describes the case of a 41-year-old pregnant woman with fetal sacral meningocele, no associated pathologies, no family history of neural tube defects, a pregnancy under folate supplementation with the aim of highlighting the importance of ultrasound in diagnosing neural tube defects.

## Case Report

**Figure 1.**
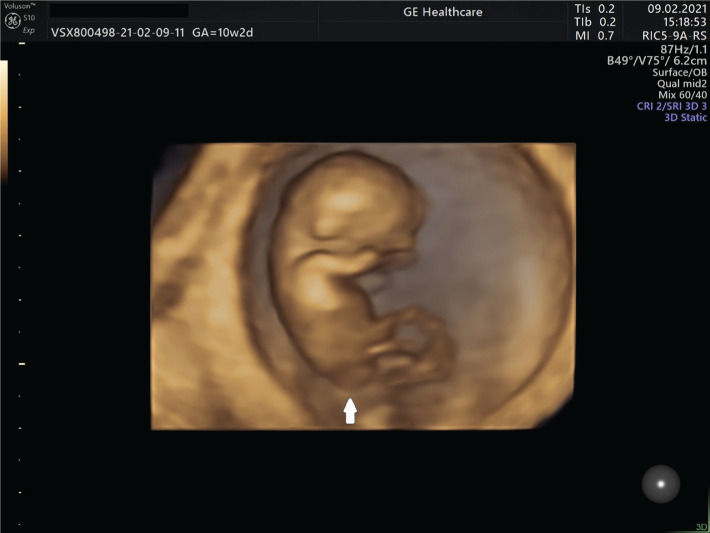
Caudal malformation at 10 gestational weeks (arrow).

A 41-year-old woman presented to our medical unit for an early diagnosis of pregnancy. Nothing abnormal was noted in the patient’s medical history. The viable intrauterine pregnancy was confirmed, and the patient was counseled to supplement the dietary intake with folate, more specifically levomefolic acid, a particular type of folic acid with a biologically active formula, to prevent neural tube defects. During the 10^th^ week of gestation, the patient presented for an ultrasound examination, and a caudal abnormality was detected with the suspicion of a neural tube defect, and it was revised at the first-trimester morphology scan (Figure 1). At the same gestational age, the patient was subjected to a non-invasive prenatal screening test, and the result was negative for the major chromosomal abnormalities.

**Figure 2.**
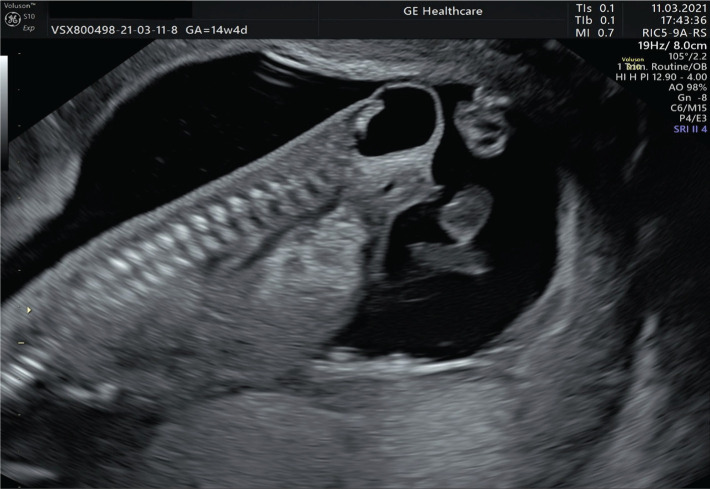
Meningocele at 14 weeks of gestation (2D ultrasonography).

Due to SARS-COV 2 (severe acute respiratory syndrome coronavirus 2) maternal infection, with minor symptomatology and the necessity of the patient to remain isolated, the patient did not present to the medical unit for the first-trimester morphology scan. The patient returned at 14 weeks and 4 days of gestation for the first-trimester morphology scan and a well delimited 1.5 cm cystic formation, with anechogenic content, without vascularization, located at the caudal extremity of the fetus, an apparent intact sacral spine being also detected (Figure 2). Interestingly, the defect appeared rather anterior, like it would arise from the anterior part of the sacrum and extended towards the perineum, giving the rarity of this atypical phenotype.

The fetus presented normal internal translucency and brain stem/brain stem-occipital bone ratio. The patient was counseled about the potential risks; generally, spina bifida with normal posterior fossa has a milder clinical presentation after birth and there is no indication for fetal surgical treatment. A second opinion evaluation sustained a cystic appearance of a coccygeal teratoma, but the Doppler study did not show the usual high vascular signal that characterizes a sacrococcigeal teratoma. The mother decided to terminate the pregnancy at 16 gestational weeks.

**Figure 3.**
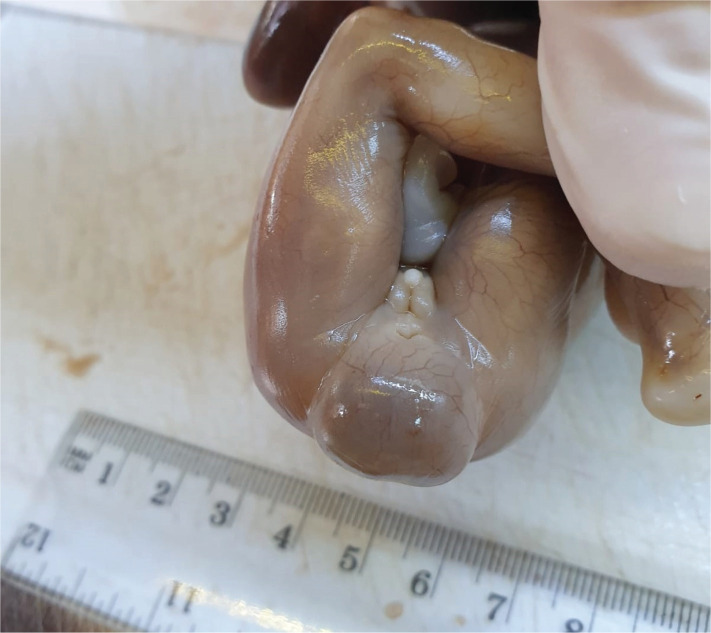
Meningocele – pathology examination (anterior view).

**Figure 4.**
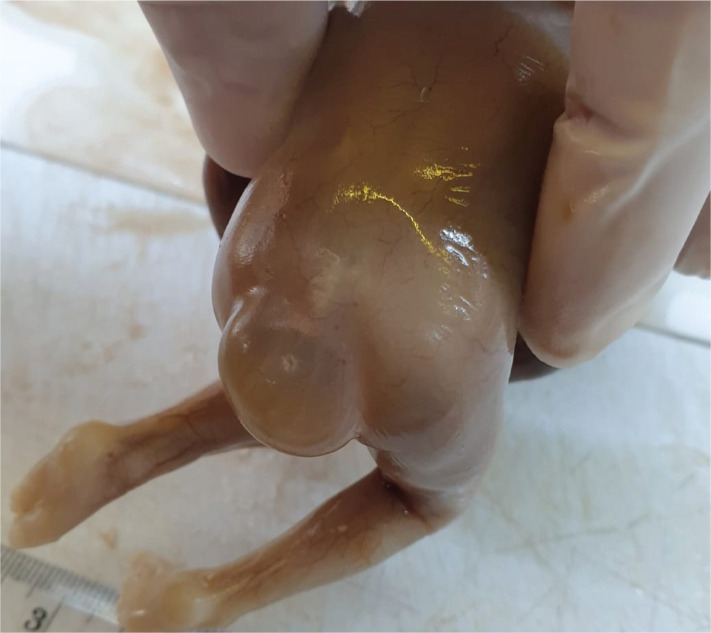
Meningocele – pathology examination (posterior view).

**Figure 5.**
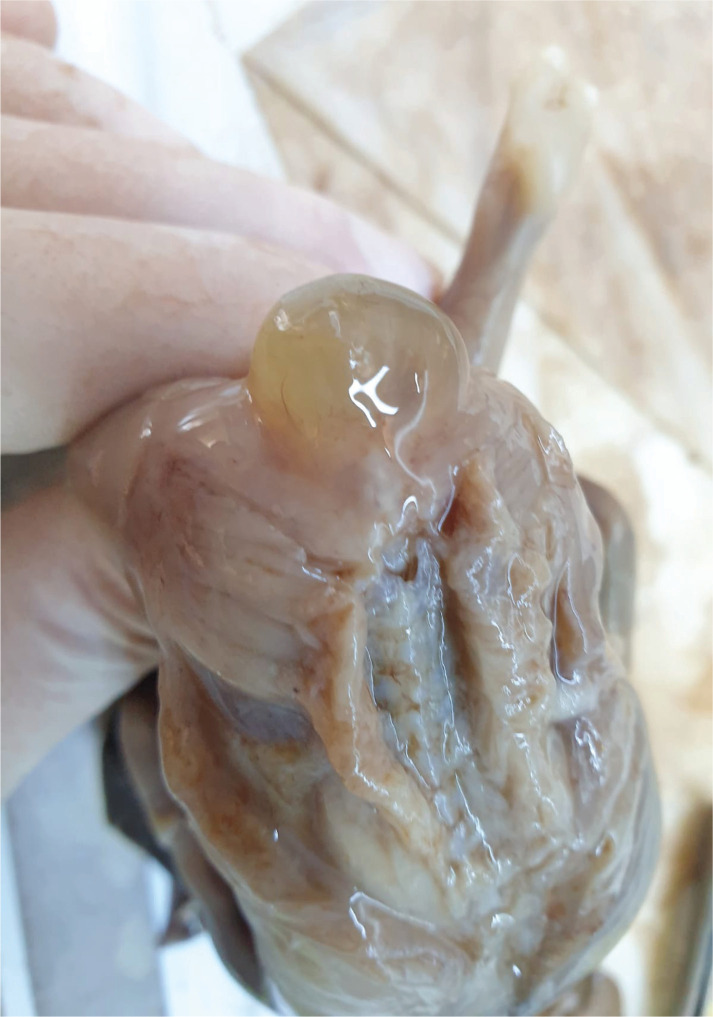
Meningocele – pathologic macroscopic aspect of the dissection.

The pathology report described the sacral malformation being surrounded by an inner meningeal tissue epithelialized cystic wall that presented an underlying connective tissue with numerous blood vessels, some of them including recent intraluminal thrombosis. The external cystic wall was covered with keratinized multilayered squamous epithelium with focal acanthosis. The pathology report supported the diagnosis of sacral meningocele (Figures 3–5).

## Discussion

The ultrasonographic diagnosis was not clear from the beginning. In our case, the differential diagnosis of meningocele was made with the cystic compound of a sacrococcygeal teratoma, which represents one of the most common congenital tumors in newborns [20].

The particularity of this case was that a neural tube defect occurred despite the prophylactic administration of folic acid during pregnancy, which represents a well-documented protection against neural tube defects in fetuses. Even though a non-invasive prenatal test was negative for the major chromosomal abnormalities, our case is an argument for the importance of the first-trimester morphology scan in detecting structural malformations that are not included in a particular syndrome, to evaluate the indirect signs of neural tube defects and to evaluate the associated spontaneous malformations. Therefore, it is of utter importance to perform the first-trimester morphology scan to evaluate the major chromosomal abnormalities markers, along with the confirmation of a normally developing fetus.

## Conclusion

In conclusion, there is a real need for trained fetal morphologists and fetal pathologists to diagnose an underlying pathology correctly, especially one as sensitive as a neural tube defect, to counsel the mother about the fetal risks and treatment options, to diagnose prematurely a possibly life-threatening condition, and subsequently to improve fetal morbidity and mortality.

## Acknowledgments

### Conflict of interest

The authors declare that there is no conflict of interest.

### Consent for publication

Informed consent to publish the data was obtained from the participant in this case report.

### Personal thanks

We would like to thank one of the reviewers for helping to increase the quality of this report through constructive criticism.

### Authorship

All the authors contributed equally to this work.
